# Fusion of Deep Convolutional Neural Networks for No-Reference Magnetic Resonance Image Quality Assessment

**DOI:** 10.3390/s21041043

**Published:** 2021-02-03

**Authors:** Igor Stępień, Rafał Obuchowicz, Adam Piórkowski, Mariusz Oszust

**Affiliations:** 1 Doctoral School of Engineering and Technical Sciences at the Rzeszow University of Technology, al. Powstancow Warszawy 12, 35-959 Rzeszow, Poland; igorkrzysztofstepien@gmail.com; 2 Department of Diagnostic Imaging, Jagiellonian University Medical College, 19 Kopernika Street, 31-501 Cracow, Poland; rafalobuchowicz@su.krakow.pl; 3 Department of Biocybernetics and Biomedical Engineering, AGH University of Science and Technology, al. Mickiewicza 30, 30-059 Cracow, Poland; pioro@agh.edu.pl; 4 Department of Computer and Control Engineering, Rzeszow University of Technology, W. Pola 2, 35-959 Rzeszow, Poland

**Keywords:** image quality assessment, deep learning, network fusion, magnetic resonance images

## Abstract

The quality of magnetic resonance images may influence the diagnosis and subsequent treatment. Therefore, in this paper, a novel no-reference (NR) magnetic resonance image quality assessment (MRIQA) method is proposed. In the approach, deep convolutional neural network architectures are fused and jointly trained to better capture the characteristics of MR images. Then, to improve the quality prediction performance, the support vector machine regression (SVR) technique is employed on the features generated by fused networks. In the paper, several promising network architectures are introduced, investigated, and experimentally compared with state-of-the-art NR-IQA methods on two representative MRIQA benchmark datasets. One of the datasets is introduced in this work. As the experimental validation reveals, the proposed fusion of networks outperforms related approaches in terms of correlation with subjective opinions of a large number of experienced radiologists.

## 1. Introduction

Image quality assessment (IQA) of magnetic resonance images (MR) plays a vital part in the diagnosis and successful treatment [1,2,3]. The IQA methods aim to provide automatic, repeatable, and accurate evaluation of images that would replace tests with human subjects. Such tests are often time-consuming, difficult to organize, and their output may depend on the considered group of participants. Therefore, the progress in the development of IQA techniques depends on the availability of assessed image databases. This is particularly important for MRIQA methods that require MR image databases with opinions of a representative number of radiologists, i.e., the databases are used for their comparison and stimulate the emergence of new approaches in the field. The IQA approaches are divided into three groups, depending on whether distortion-free images are used: full-reference (FR), reduced-reference (RR), and no-reference (NR). The availability of unaltered, distortion-free, reference images is a basis for their differentiation. Nevertheless, such pristine images, or their partial characteristics, are unavailable for MR images, limiting the practical application of FR and RR methods. Therefore, the NR MRIQA measures are highly desired, while FR and RR approaches are mostly employed for artificially distorted, high-quality MR images.

There are several approaches to the FR medical IQA [4]. Among them, the Peak Signal-to-Noise Ratio (PSNR) is the most popular. However, it might not be accurate enough to give a proper measure between distorted and reference images, concerning their characteristics and known inability of the PSNR to reliably reflect human subjective opinions. In the assessment of medical images, its derivative approaches, i.e., the signal-to-noise ratio (SNR) and contrast-to-noise ratio (CNR) [1], are often used. Furthermore, some early methods adapt solutions from the IQA of natural images [5], train them on images assessed by the SNR [2], or add additional features to characterize MR images [6]. Some approaches employ the entropy of local intensity extrema [7] or specific image filtering to facilitate the usage of local features [8]. Other works are devoted to a binary classification of noisy MR images [9,10] or for classification of images with prior detection of selected distortion types [11,12].

In this paper, taking into account the lack of NR IQA measures devoted to MR images in the literature, a novel NR method is proposed in which deep learning architectures are fused and the transfer-learning process is jointly performed. The resulted fusion allows the network to better characterize distorted MR images due to the diverse backgrounds of employed architectures. Furthermore, to improve the quality prediction of the approach, the SVR is used on features extracted from fused networks. As most of the databases used in the literature are not publicly available, contain artificially distorted images, and/or were assessed by a few radiologists, in this paper, a novel IQA MRI database with images assessed by a large number of radiologists is introduced.

The main contributions of this work are as follows: (i) Fusion of deep convolutional network architectures for MRIQA. (ii) The usage of the SVR with features obtained in joint transfer learning of networks to improve the performance of the method. (iii) Novel large IQA database of MR images assessed by a large number of radiologists. (iv) Extensive evaluation of the numerous deep learning architectures and related techniques.

The remainder of the paper is organized as follows. In Section 2, previous work on NR-IQA is reviewed, while in Section 3, the proposed approach is introduced. Then, in Section 4, the experimental validation of the method and related techniques is presented. Finally, Section 5 concludes the paper and indicates future directions of the research.

## 2. Related Work

The introduced method belongs to the category of NR techniques that use a deep learning approach to predict the quality of assessed images. However, before such approaches were possible for the IQA of natural images, many handcrafted IQA measures were proposed. For example, the method of Moorthy and Bovik [13] employs a two-stage framework in which distortion type is predicted and used for the quality evaluation. A framework in which a probabilistic model with DTC-based natural scene statistics (NSS) is trained was proposed by Saad et al. [14]. Then, the popular BRISQUE technique [15] was introduced, which uses the training of the Generalized Gaussian Distribution (GGD) with Mean Substracted Contrast Normalization (MSCN) coefficients. The Gabor features and the soft-assignment coding with the max-pooling are employed in the work of Ye et al. [16]. In the High Order Statistics Aggregation (HOSA) [17] method, low and high order statistics for the description of normalized image patches obtained from codebook using the soft assignment is presented. In the method, the codebook was obtained with the k-means approach. As gradient-based features can effectively describe distorted images, many approaches use them for quality prediction. They employ global distributions of gradient magnitude maps [18], relative gradient orientations or magnitude [19], and local gradient orientations captured by Histogram of Oriented Gradient (HOG) technique for variously defined neighborhoods [20]. A histogram of local binary patterns (LBP) characterizing a gradient map of an image is used in the GWHGLBP approach [21]. In the NOREQI [22] measure, an image is filtered with gradient operators and described using speeded-up robust feature (SURF) descriptor. Then, the descriptors are characterized by typical statistics. The joint statistics of the gradient magnitude map and the Laplacian of Gaussian (LOG) response are used to characterize images in the GM-LOG technique [18].

Most learning-based NR-IQA techniques devoted to natural images employ the SVR method to create a quality model. However, some methods do not require training. For example, in the Integrated Local Natural Image Quality Evaluator (IL-NIQE) [23], natural image statistics derived from multiple cues are modeled by the multivariate Gaussian model and used for the quality prediction without additional training step. In the BPRI [24], a pseudo-reference image is created and compared with the assessed image using quality metrics that measure blockiness, sharpness, and noisiness.

In recent years, more complex IQA approaches have been introduced that use deep neural network architectures. They do not contain a separate image description and prediction stages. However, their training requires a large amount of data or an adaptation of architectures developed for computer vision tasks not related to the IQA. Some of the early models address those challenges by using image patches [25,26], training with scores generated by FR-measures or [26,27], or fine-tuning of popular networks [28].

Considering the quality assessment of MR images, the number of approaches is much less diverse. Here, only several works have been published, revealing the lack of successful techniques and the scarcity of the IQA MRI benchmarks that could be used to stimulate their development. Furthermore, most clinical applications use the SNR and CNR [1] measures to assess images or calibrate scanners. However, they require an indication of disjoint image regions with noise and tissue, despite providing an inferior quality evaluation of images in comparison with modern methods. Some of NR IQA measures designed for the assessment of MR images adapt solutions devoted to natural images. For example, Chow and Rajagopal [5] trained the BRISQUE on MR images, Yu et al. [2] used SNR scores to train BRISQUE and three other IQA methods, while Jang et al. [6] used MSCN multidirectional-filtered coefficients. In the work of Esteban et al. [9], image quality was not predicted but binarily classified based on a set of simple measures. Taking into account the inclusion of neural network architectures for processing MR images, Kustner et al. [11] and Sujit et al. [12] detected motion artifacts and performed binary classification of structural brain images, respectively. Volumetric and artifact-specific features were used by Pizarro et al. [10] to train the SVM classifier. In previous authors’ works on the MRIQA, the entropy of local intensity extrema was used for direct quality prediction [7] or high-boost filtering followed by the detection and description of local features [8] was used with an SVR-based quality model.

Taking into account the lack of deep learning architectures for the MRIQA, it can be stated that their effectiveness remains largely uninvestigated, and the introduction of their effective fusion can be seen as a promising area of research.

## 3. Proposed Method

In the proposed approach, a fusion of deep network architectures is considered. Such architectures are mostly devoted to image recognition tasks and were propagated to other areas of computer vision [29]. Among popular deep learning networks, the approach of Simonyan and Zisserman [30] (VGG) uses 3×3 convolutional filters and achieves outstanding performance at the ImageNet Large Scale Visual Recognition Competition (ILSVRC) 2014 competition. Another solution, Resnet [31], introduces a residual learning framework, with a shortcut connection between layers to address the overfitting experienced by the VGG. With the same purpose, Szegedy et al. [32,33] introduced the Inception module in the GoogLeNet model. In other works, Howard et al. [34] created Mobilenet aiming to reduce the computational costs, or Huang et al. [35], in the DenseNet, used network layers with inputs from all preceding layers.

Deep learning models were also used for the IQA of natural images [26,36,37,38,39]. Most of such adaptations employ transfer learning, making the networks aware of domain-specific characteristics. Therefore, in this study, a similar approach was applied at the beginning of the research. Thus, adapted single models can be seen as counterparts of the first approaches with deep learning models to the IQA of natural images. However, the performance of a single network turned out to be insufficient to provide superior performance in the MRIQA task (see Section 4.6). Therefore, the approach introduced in this paper considers an internal fusion of networks, assuming that the fusion of different network types can capture characteristics of MR images, leading to outstanding IQA accuracy across the benchmark datasets.

Considering an image $I_{n}$ that belongs to a set of *N* training images, n=1,⋯,N, each of which is associated with subjective score $q_{n}^{s}$ obtained in tests with human subjects. Note that the subjective scores are denoted as Mean Opinion Scores (MOS) or Difference MOS (DMOS). The input image $I=R^{h\times w\times c}$, where *h*, *w*, and *c* corresponds to the height, width, and the number of channels, respectively, is processed by the network. The network often consists of stacked modules of convolutional and pooling layers, and several fully connected layers. The convolutional layers extract features from earlier layers. This can be written as $O_{k}=f\left(W_{k}⊛I\right)$, where $W_{k}$ denotes a *k*-th filter or weights, ${}^{‘}{⊛}^{’}$ is convolutional operator, and f is the nonlinear activation function, often represented by rectified linear units (ReLUs). The pooling layers reduce feature maps, introducing average or maximum values of inputs to the next layers. For example, the max pooling $O_{k}\left(a,b\right)=max\left(I_{k}\left(i,j\right)\right)$, where the (i,j)∈P(a,b) denotes location of the element in the pooled region P(a,b). The fully connected layers are used to interpret features and provide high-level problem representations. Their outputs are further processed by the softmax or regression layer for the classification or regression problems, respectively.

### Network Fusion

**Figure 1:**
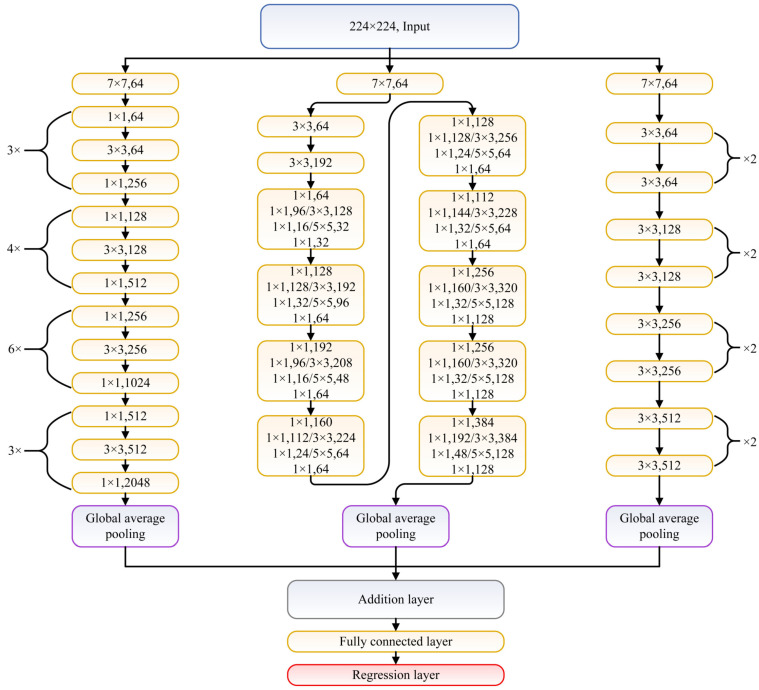
Example of fusion network composed of ResNet-50 (**left**), GoogleNet (**center**), and ResNet-18 (**right**).

As the number of images in MRIQA benchmarks is not large enough for efficient training of considered network architectures or their fusions, in this study, transfer learning [40] is applied instead of learning from scratch. The considered networks are pretrained on ImageNet dataset and classify images into 1000 categories. However, the IQA task requires solving the regression problem, which forces the modification of the architecture of the network towards the quality prediction purpose. Therefore, in this study, the last three network layers of each used network, configured for 1000 classes, are replaced with a fully connected layer and the regression layer. Note that the replacement is performed regarding all network architectures, either single or fused. Here, the networks share the inputs and are connected to each other with a feature concatenation layer that adds outputs from multiple layers in an element-wise manner. If needed, the input image is resized to match the input size of a network. As layers responsible for average pooling remain in each network architecture after the removal of the part associated with image classification, they are used as inputs to the concatenation (addition) layer. An exemplary fusion of ResNet-18, ResNet-50, and GoogLeNet is presented in Figure 1. In the network graph, each network is represented by connected sets of convolution layers (yellow blocks). Among the last elements in each network are the global average pooling layer, addition layer that fuses their outputs, fully connected layer, and regression layer.

For the training of the resulted network architecture, *N* images are used. However, as MR images are often 2-dimensional 16-bit matrices, they are concatenated to form three channels (*c* = 3) to facilitate processing by the pretrained networks. To estimate network parameters, the half Mean Squared Error (MSE) loss function *L* is applied. Considering that $Q^{o}=\left(q_{1}^{o},\cdots ,q_{N}^{o}\right)$ is the vector of objective scores and $Q^{s}=\left(q_{1}^{s},\cdots ,q_{N}^{s}\right)$ represents subjective scores, *L* is calculated as

(1) $$L\left(Q^{s},Q^{o}\right)=\frac{1}{N}\sum_{n=1}^{N}{\left(q_{n}^{s}-q_{n}^{o}\right)}^{2}.$$

Typically, transfer learning of the network assumes freezing the original layers to prevent back-propagation of errors. However, as in this study MR images are processed and have different characteristics from natural images, the weights of fused networks were modified using a small learning rate.

Once the training of the network is finished, a second step of the approach is executed in which concatenated feature vectors (see the addition layer in Figure 1) are used as an input *x* to the SVR module to obtain a quality prediction model, $x=x_{net_{1}}⊕x_{net_{2}}⊕\cdots ⊕x_{net_{M}}$, where ⊕ is the concatenation operator and *M* is the number of fused deep learning architectures.

The SVR technique is commonly used to map perceptual features to MOS. In this paper, the ε-SVR is employed for training the regression model. Given training data $(X,Q_{s})=$ {$\left(x_{1},q_{1}^{s}\right)$, …, $\left(x_{N},q_{N}^{s}\right)$}, where $x_{n}$ is the feature vector and $q_{n}^{s}$ is its MOS, a function f(x)=〈ω,*x*〉 + *b* is determined in which 〈·,·〉 denotes the inner product, ω is the weight vector, and *b* is a bias parameter. Introducing the slack variables $\xi_{n}$ and $\xi_{n}^{*}$, ω and *b* can be computed by solving the following optimization problem,

(2) $$\begin{array}{cc} & \mathrm{minimize}\;\frac{1}{2}{∥\omega ∥}^{2}+C\sum_{n=1}^{N}\left(\xi_{n}+\xi_{n}^{*}\right)\\ \; & subject\;to\left\{\begin{array}{c} \langle \omega ,x_{n}\rangle -\left(q_{n}^{s}-b\right)\leq \varepsilon +\xi_{n}\\ q_{n}^{s}-b-\langle \omega ,x_{n}\rangle \leq \varepsilon +\xi_{n}^{*}\\ \xi_{n},\xi_{n}^{*}\geq 0, \end{array}\right. \end{array}$$

where *C* is a the constant parameter to balance ω and the slack variables. The $\omega =\sum_{n=1}^{N}t_{n}x_{n}$, where $t_{n}$ is a combination coefficient. Usually, in the first step, the input feature vector is mapped into a high-dimensional feature space Φ(x), and then the regression function is obtained:

(3) $$\begin{array}{cc} f(x)= & \langle \sum_{n=1}^{N}t_{n}\Phi \left(x_{n}\right),\Phi \left(x\right)\rangle +b\\ = & \sum_{n=1}^{N}t_{n}\langle \Phi \left(x_{n}\right),\Phi \left(x\right)\rangle +b. \end{array}$$

The inner product $\langle \Phi \left(x_{n}\right),$Φ(x)〉 can be written as a kernel function $c(x_{n},x)$. Therefore,

(4) $$f\left(x\right)=\sum_{n=1}^{N}t_{n}c\left(x_{n},x\right)+b.$$

The radial base function (RBF) is often used as c, $c\left(x_{n},x\right)=exp(-\gamma (|x_{n}-{x|)}^{2})$, where the γ is the precision parameter [18].

**Figure 2:**
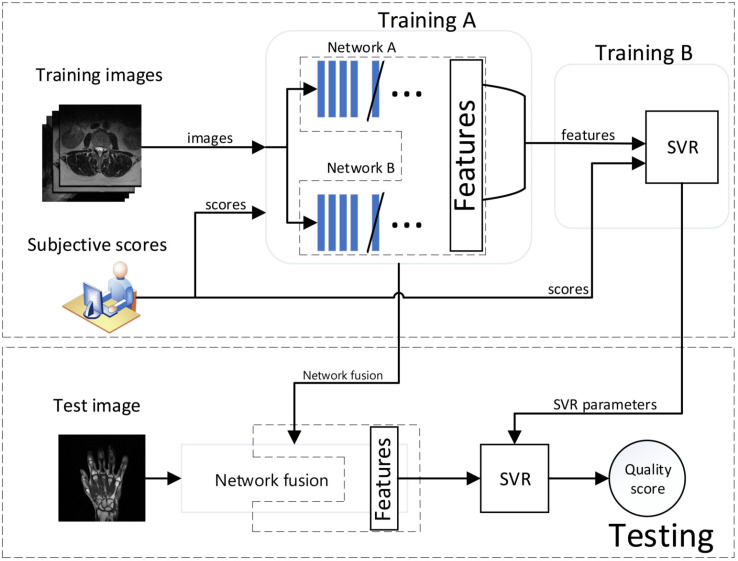
Block diagram of the proposed approach.

The main computational steps of the approach are shown in Figure 2. As it can be seen, networks are fused and trained together (*training A*) to capture MR-specific characteristics. Then, the SVR module is trained with concatenated feature maps (*training B*) to obtain the quality model used in the prediction.

In this paper, the following networks are considered in the fusion: DenseNet-201 [35], GoogLeNet [32], Inception-v3 [33], MobileNet-V2 [34], ResNet-101 [31], ResNet-18 [31], and ResNet-50 [31]. The networks process 224 × 224 images (instead of Inception-v3 that works with 299 × 299 images). The ResNet employs 18, 50, or 101 layers, while GoogLeNet, Inception-v3, MobileNet-V2, and DenseNet-201, use 22, 48, 53, and 201 layers, respectively.

**Figure 3:**
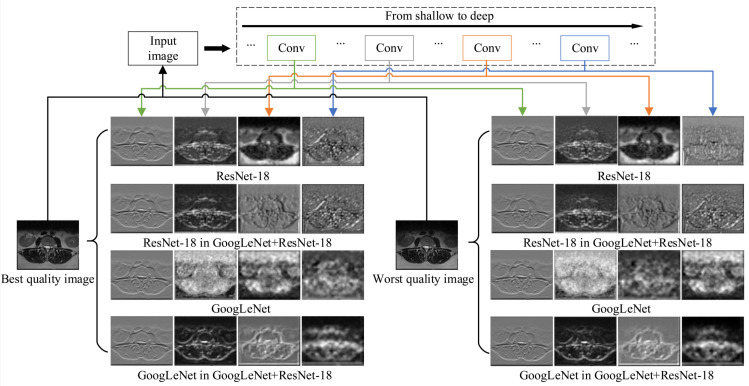
Visualization of features at different layers for the best and worst quality MR images. The GoogLeNet, ResNet-18, and their fusion are shown.

To further justify the need for network fusion proposed in this paper and show its sensitivity to distortions, a visualization of exemplary features processed by single and fused networks using DeepDream (https://www.tensorflow.org/tutorials/generative/deepdream (accessed on 23 December 2020)) technique is provided in Figure 3. The technique is often used to show what a given network has learned at a given stage. In the experiment, the best and worst quality images of the same body part were used. As presented, the features in fused networks distinctively respond to distortions that are propagated through the architecture. Furthermore, their features seem affected by the existence of another network in the training which modifies their response comparing to single architectures. Interestingly, the features of GoogLeNet or ResNet in the GoogLeNet+ResNet-18 fusion are more similar to each other than to features from single network architectures. This can be attributed to joint transfer learning. The fusion exhibits a different response to different distortion severity. Consequently, it can be assumed that the quality prediction model based on the fusion would be able to reliably evaluate MR images of different quality.

## 4. Results and Discussion

### 4.1. Experimental Data

The proposed approach is evaluated on two MRIQA benchmark datasets. The first dataset, denoted for convenience as *DB1*, contains 70 MR images [8], while the second one (*DB2*) has been created for the needs of this study and contains 240 MR images.

**Figure 4:**
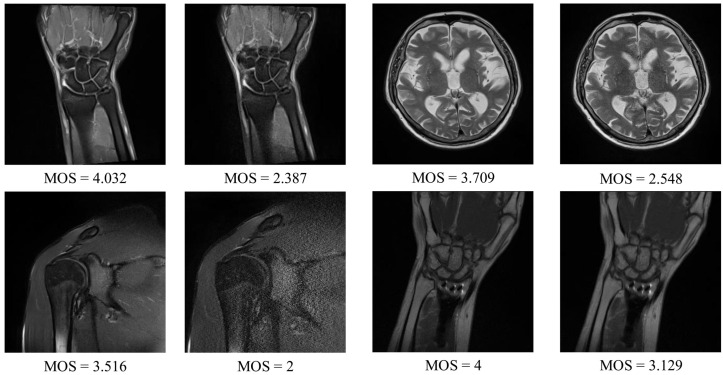
DB1 benchmark: Exemplary magnetic resonance (MR) images and their subjective scores.

The DB1 benchmark contains images selected from 1.5T MR T2-weighted sagittal sequences: the spine (14 images), knee (14), shoulder (16), brain (8), wrist (6), hip (4), pelvis (4), elbow (2), and ankle (2). The collection consists of images captured under different conditions affecting the image quality (IPAT software to made GeneRalized Autocalibrating Partially Parallel Acquisitions (GRAPPA); GRAPPA3 [41]). Apart from the images, the benchmark contains the MOS, ranging from 1 to 5, which was obtained in tests with a group of radiologists [8]. The resolution of images in the dataset is between 192 × 320 and 512 × 512. Exemplary images that can be found in the DB1 are shown in Figure 4.

**Figure 5:**
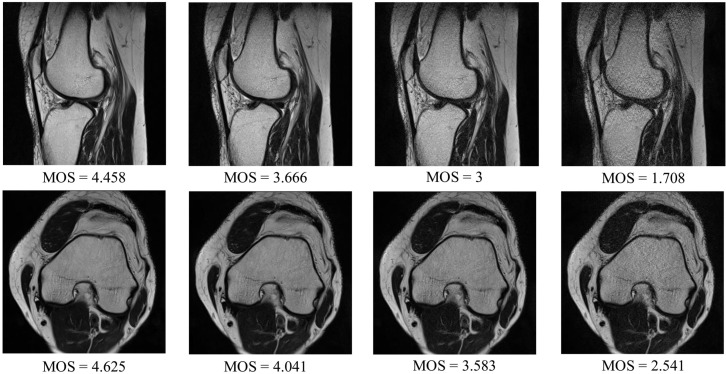
Introduced MRIQA benchmark (DB2): Exemplary MR images and their subjective scores.

As the DB1 collection is relatively small and databases that can be found in the literature were created for different purposes, are not available, or were assessed by a small number of radiologists, a novel dataset has been introduced in this study. The DB2 collection contains T2 weighted MR images acquired during routine diagnostic exams of the shoulders, knees, and cervical and lumbar spine. Patients aged 29–51 yo participated in the study. Siemens Essensa 1.5 Tesla scanner equipped with table coils, 6-channel—knee and 4-channel—shoulder coils, were used to obtain two-dimensional images in axial, coronal, and sagittal planes. The gradient strength and a slew rate of the scanner were set to 30 mT/m, and 100 T/m/s, respectively. The following parameters were also used: the echo time TE ranged from 3060 up to 6040 ms, repetition time TR (77 to 101 ms), phase oversampling of 20, distance factor of 30, and flip angle of 150°. The dataset was made on matrices from 192 × 320 to 512 × 512, using a voxel of nonisotropic resolution at 0.8 mm × 0.8 mm × 3 mm. To obtain images of different quality in a controlled way, the parallel imaging technique was applied (Siemens IPAT software) [41], reducing the number of echoes. The parallel imaging shortens the acquisition time [42] as it is commonly employed to increase patient comfort. However, in this study, it was applied to obtain degraded images during the routine imaging process. The T2 sequences were obtained using the GRAPPA approach, repeated in four modes with gradually increased severity of echo reduction. Here, GRAPPA1, GRAPPA2, GRAPPA3, and GRAPPA4 were consecutively applied, resulting in up to a 4-minute increase of total patient examination time. Finally, 30%, 40%, 60%, and 80% of the signals were lost with GRAPPA 1–4, respectively [43,44]. Obtained images were anonymized and saved ensuring the highest standards of personal data safety. Then, the DB2 was created with images of different patients. The subset of 30 exams was further investigated: knee (images of eight patients), shoulder (10 patients), cervical spine (three patients), and lumbar spine (nine patients). Then, from sequences of a better and worse quality associated to a given patient, two images per sequence were automatically selected. The selected scans were located at 1/3 and 2/3 of the length of each sequence. Once the 240 images were selected for subjective tests, a large group of 24 radiologists with more than 6 years of experience in MR study reading was invited for the assessment of their quality. The group was gathered in a room with limited access to daylight, reflecting typical conditions of such examination. Images of the different quality were displayed in pairs and presented to radiologists for 30 s (each pair) on Eizo Radi-Force high-resolution monitors (2600×1800) connected to dedicated graphic cards. Each radiologist was introduced to the assessment procedure and assigned scores from 1 to 5 to each evaluated image on paper evaluation cards. In the assessment, a higher grade reflects a better quality of an image. Then, subjective scores were processed and averaged to obtain MOS. Exemplary images from the DB2 are presented in Figure 5.

### 4.2. Evaluation Methodology

Image quality assessment techniques are evaluated and compared on benchmark datasets using four performance criteria [45]: Spearman rank-order correlation coefficient (SRCC), Kendall rank-order correlation coefficient (KRCC), Pearson linear correlation coefficient (PLCC), and Root Mean Square Error (RMSE). The higher SRCC, KRCC, and PLCC, and lower RMSE, the better output of objective IQA approach. The calculation of the PLCC (Equation (5)) and RMSE (Equation (6)) require a nonlinear mapping of objective scores $Q^{o}$ fitted with a regression model, $Q^{o}$, and subjective opinions $Q^{s}$. The model employed for the mapping is expressed as $Q^{o}=\beta_{1}\left(\frac{1}{2}-\frac{1}{1+\exp(\beta_{2}\left(Q^{o}-\beta_{3}\right))}\right)+\beta_{4}Q^{o}+\beta_{5}$, where $\beta =[\beta_{1},\beta_{2},\cdots ,\beta_{5}]$ [45]. The PLCC is calculated as

(5) $$\mathrm{PLCC}=\frac{{{\overset{¯}{Q}}^{o}}^{T}{\bar{Q}}^{s}}{\sqrt{{{\overset{¯}{Q}}^{o}}^{T}{\overset{¯}{Q}}^{o}{{\bar{Q}}^{s}}^{T}{\bar{Q}}^{s}}},$$

where ${\overset{¯}{Q}}^{o}$ and ${\bar{Q}}^{s}$ are mean-removed vectors. The RMSE is calculated as

(6) $$\mathrm{RMSE}=\sqrt{\frac{{\left(Q^{o}-Q^{s}\right)}^{T}\left(Q^{o}-Q^{s}\right)}{m}},$$

where *m* is the total number of images. The SRCC is defined as

(7) $$\mathrm{SRCC}=1-\frac{6\sum_{i=1}^{m}d_{i}^{2}}{m(m^{2}-1)},$$

where $d_{i}$ is the difference between *i*-th image in $Q^{o}$ and $Q^{s}$, i=1,2,⋯,m. Consequently, the KRCC calculated as

(8) $$\mathrm{KRCC}=\frac{m_{c}-m_{d}}{0.5m(m-1)},$$

where $m_{c}$ is the number of concordant pairs in the dataset, and $m_{d}$ denotes the number of discordant pairs.

As the proposed approach should be trained to obtain a quality model for the prediction, a widely accepted protocol for the evaluation of related methods is used in which 80% of randomly selected images of the dataset are selected for the training and the remaining 20% of images test the approach [16,24]. Both image subsets are disjoint based on the experiment that leads to the acquisition of images of a given body part. Then, to avoid bias, the performance of an NR method is reported in terms of the median values of SRCC, KRCC, PCC, and RMSE over 10 training–testing iterations [27,46].

### 4.3. Comparative Evaluation

The introduced approach is represented by three fusion models: Resnet-50_GoogLeNet _ResNet-18 (R50GR18), ResNet-50_GoogLeNet_MobileNet-V2 (R18GR50M), and MobileNet-V2_ResNet-50 (MR50). They are experimentally compared with 17 state-of-the-art techniques: NFERM [47], SEER [20], DEEPIQ [27], MEON [46], SNRTOI [48], NOREQI [22], BPRI [24], HOSA [17], NOMRIQA [8], IL-NIQE [23], GM-LOG [18], GWHGLBP [21], BRISQUE [15], SISBLIM [49], metricQ [50], SINDEX [51], and ENMIQA [7]. The NOMRIQA, ENMIQA, and SNRTOI are designed for MR images, whereas DEEPIQ and MEON are deep learning approaches devoted to natural images. Interestingly, as Chow and Rajagopal [5] trained the BRISQUE on MR images in their approach, the BRISQUE in this study, as well as other methods trained on considered benchmark databases, can be seen as adaptations to the MR domain.

For a fair comparison, all methods were run in Matlab with their default parameters, while the SVR parameters [52] were determined aiming at their best quality prediction performance. NR techniques that process color images were assessing MR images concatenated to form three channels. For the training of the proposed fusion architectures, stochastic gradient descent with momentum (SGDM) was used with a learning rate of $10^{-4}$, mini-batch size of 32, and 5 epochs. Furthermore, as the number of images in the first dataset is relatively low (70), data augmentation was employed in which each distorted image was rotated up to 360${}^{\circ }$ with the step of $3^{\circ }$. The approaches were run in Matlab R2020b, Windows 10, on PC with i7-7700k CPU, 32GB RAM, and GTX 1080 Ti graphic card.

The results for both databases are presented in Table 1. Their analysis indicates that the proposed network fusion techniques outperform the state-of-the-art approaches. Specifically, the R50GR18 (composed of three network architectures) and MR50 (fusing two networks) obtained the best SRCC and KRCC performances for the first database, outperforming the recently introduced NOMRIQA. This is also confirmed by the results for the remaining criteria, i.e., PLCC and RMSE. It is worth noticing that NR MRIQA methods that do not require training, ENMIQA and SNRTOI, produce poorly correlated quality opinions while compared with outputs of learning-based approaches as they are not equipped with various perceptual features and powerful machine learning algorithms that efficiently map them with subjective scores.

For the database introduced in this paper, DB2, the R18GR50M, and R50GR18 present superior performance, followed by the SEER and MR50. Here, more techniques obtain better results in comparison to those obtained for the DB1 due to the larger representation of distorted images in the dataset. The methods designed for MR images—ENMIQA and NOMRIQA—are outperformed by well-established learning methods devoted to natural images (SEER, HOSA, GM-LOG, or NFERM). Deep learning models—MEON and DEEPIQ—were pretrained by their authors and do not capture characteristics of MR images, leading to inferior prediction accuracy. The overall results, averaging criteria over both databases, indicate the superiority of introduced fusion approaches. In this case, the NOMRIQA is fourth in terms of the SRCC values, followed by NFERM and GWHGLBP after a large performance gap.

**Figure 6:**
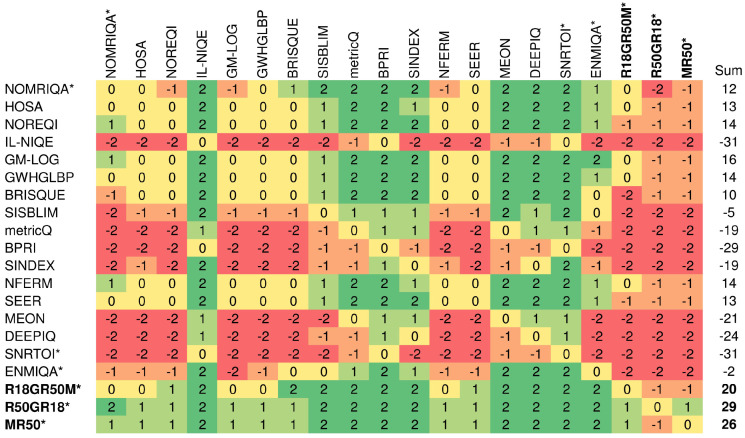
Summary of statistical significance tests on both databases. The approach designed for the evaluation of MR images is indicated with *. The names of the best three methods and sums of their scores are written in bold.

To compare relative differences between methods and determine whether they are statistically significant, the Wilcoxon rank-sum test is used. The test measures the equivalence of the median values of independent samples with a 5% significance level [20]. Here, the SRCC values are taken into consideration. In the experiment, the method with a significantly greater SRCC median obtained the score of “1”. Consequently, the worse and indistinguishable method obtained “−1” and “0”, respectively. Finally, scores were added and displayed in cells in Figure 6 to characterize methods in rows. The figure also contains sums of scores to indicate globally best approaches. As reported, all three introduced fusion architectures offer promising and stable performance across both datasets, outperforming the remaining approaches. Other learning-based methods designed for the MRIQA, i.e., ENMIQA or NOMRIQA, exhibit inferior relative performance in comparison to the proposed models and methods with rich image representations (GM-LOG, SEER, or NFERM).

### 4.4. Computational Complexity

The computational complexity of methods, reflected by the average time taken to assess an image from DB2, was also investigated (Table 2). The time spent on extracting and predicting the quality by a method based on the fusion of networks depends on the number of networks. However, the proposed models are of moderate complexity, being on par with the fastest and more reliable approaches. Further reduction of the computation time can be achieved by a parallel feature extraction process or providing a native implementation (e.g., C++).

### 4.5. Cross—Database Experiments

The performances of R18GR50M, R50GR18, and MR50 are compared with those of related IQA methods in the cross-database experiment. In the experiment, learning-based methods are trained on one database and tested on another. The methods that do not require training are only tested on the second database. The obtained results are shown in Table 3. As reported, the introduced fusion models outperform other techniques and exhibit stable prediction accuracy. Here, the method for IQA of MR images, NOMRIQA, is close to the proposed architectures. The values of performance indices of fusion models trained on the small DB1 and tested on much larger DB2 are only several percent lower than values reported for the DB2 in the first experiment (see Table 1). This confirms their capability of successful extraction of MR image characteristics needed for the quality prediction. Overall, all three introduced architectures provide superior performance, followed by NOMRIQA and SEER.

### 4.6. Ablation Tests

As in the literature many different network architectures have been introduced, in this section, several proposing fusion approaches are reported and discussed. Furthermore, the inclusion of the SVR method is also supported experimentally to show that it improves the results of the networks. In experiments, single deep learning networks or their fusions were considered. The following fusions took part in the study: ResNet-50_GoogLeNet_MobileNet-V2 (R18GR50M), GoogLeNet_DenseNet-201 (GD201), GoogLeNet_MobileNet-V2 (GM), GoogLeNet_ResNet-101 (GR101), GoogLeNet_ResNet-18 (GR18), GoogLeNet_ResNet-50 (GR50), MobileNet-V2_ResNet-101 (MR101), MobileNet-V2_ResNet-18 (MR18), MobileNet-V2_ResNet-50 (MR50), ResNet-50_GoogLeNet_ResNet-18 (R50GR18), ResNet-50_Inception-V3 (R50Iv3), ResNet-50_ResNet-101 (R50R101), and ResNet-50_ResNet-18 (R50R18).

**Figure 7:**
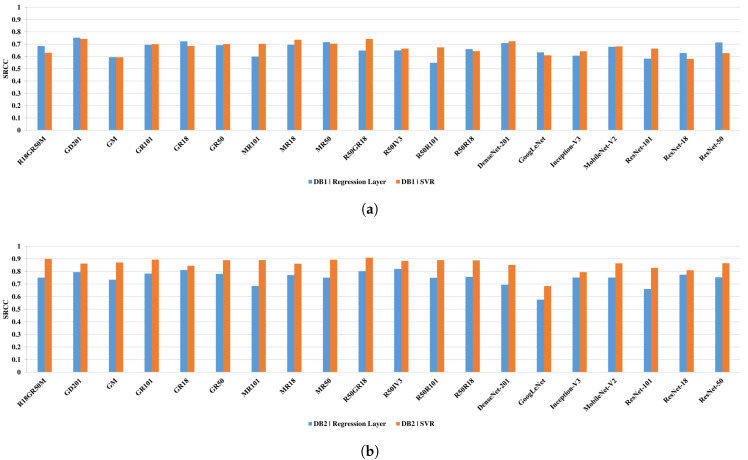
Spearman rank-order correlation coefficient (SRCC) performance of compared single and fused networks for the DB1 (**a**) and DB2 (**b**) databases. The results involve quality prediction performed by networks or support vector machine regression (SVR) modules.

The results presented in Figure 7 reveal that the usage of the SVR module improves the results of the half networks for the DB1 and in all cases for the DB2. Interestingly, most network architectures outperform other state-of-the-art IQA methods on both databases (see Table 1), showing that they can be successfully used for the quality prediction of MR images. However, network architectures that are based on the proposed fusion of single models offer better performance than it can be seen for single networks. Among single architectures, ResNet-50, MobileNet-V2, and DenseNet-201 yield promising results. Therefore, two of them—ResNet-50 and MobileNet-V2—were fused together with ResNet-18 and GoogLeNet obtaining the best performing fusion architectures: (R18GR50M, R18GR50M, and MR50). Here, the fusion with the worst-performing GoogLeNet seems beneficial as its features turned out to be complementary with those of other networks.

## 5. Conclusions

In this study, a novel no-reference image quality assessment approach for automatic quality prediction of MR images has been presented. In the approach, deep learning architectures are fused, suited to the regression problem, and, after joint transfer learning, their concatenated feature maps are used for quality prediction with the SVR technique. The usage of two or more network architectures, the way they are fused, and their application to the no-reference IQA of MR images are among contributions of this work. Furthermore, several promising fusion models are proposed and investigated as well as a novel dataset for the development and evaluation of NR methods. The dataset contains 240 distorted images assessed by a large number of experienced radiologists. The comprehensive experimental evaluation of fusion models against 17 state-of-the-art NR techniques, including methods designed for NR IQA of MR images, reveals the superiority of the presented approach in terms of typical performance criteria.

Future work will focus on the investigation of alternative network fusion approaches or developing NR measures for IQA of medical images with different specificity, e.g., CT or RTG.

## Figures and Tables

**Table 1:** Performance comparison of twenty evaluated methods on both datasets.

Method	DB1				DB2				Overall			
	SRCC	KRCC	PLCC	RMSE	SRCC	KRCC	PLCC	RMSE	SRCC	KRCC	PLCC	RMSE
NOMRIQA *	**0.7030**	**0.5527**	0.7978	0.4322	0.8040	0.6087	0.8737	0.4605	0.7535	0.5807	0.8358	0.4464
HOSA	0.4804	0.3909	0.6997	0.5318	0.8756	0.7052	**0.9276**	**0.3388**	0.6780	0.5481	0.8137	0.4353
NOREQI	0.4359	0.2922	**0.8045**	**0.4182**	0.8675	0.6984	0.9072	0.3833	0.6517	0.4953	0.8559	0.4008
IL-NIQE	0.1695	0.1275	0.3619	0.6674	0.1197	0.0836	0.3090	0.8821	0.1446	0.1056	0.3355	0.7748
GM-LOG	0.4673	0.3424	0.6515	0.4779	0.8854	0.7123	0.9010	0.4091	0.6764	0.5274	0.7763	0.4435
GWHGLBP	0.5075	0.3935	0.6886	0.5257	0.8726	0.6927	0.8947	0.4080	0.6901	0.5431	0.7917	0.4669
BRISQUE	0.4610	0.3648	0.6100	0.5311	0.8544	0.6738	0.8951	0.4076	0.6577	0.5193	0.7526	0.4694
SISBLIM	0.3976	0.2776	0.6240	0.5449	0.7216	0.5419	0.7592	0.6047	0.5596	0.4098	0.6916	0.5748
metricQ	0.2596	0.1657	0.2792	0.6709	0.5066	0.3701	0.5227	0.7791	0.3831	0.2679	0.4010	0.7250
BPRI	0.2412	0.1890	0.4785	0.5756	0.1317	0.0973	0.4928	0.7883	0.1865	0.1432	0.4857	0.6820
SINDEX	0.2939	0.2112	0.3243	0.7034	0.2673	0.1933	0.3185	0.8874	0.2806	0.2023	0.3214	0.7954
NFERM	0.5073	0.4091	0.7662	0.4491	0.8833	0.7087	0.9157	0.3872	0.6953	0.5589	0.8410	0.4182
SEER	0.4776	0.3574	0.7108	0.5267	**0.8938**	**0.7335**	0.9196	0.3594	0.6857	0.5455	0.8152	0.4431
MEON	0.2518	0.1879	0.3439	0.6428	0.5851	0.4001	0.6194	0.7426	0.4185	0.2940	0.4817	0.6927
DEEPIQ	0.1133	0.0827	0.5902	0.5707	0.2837	0.2078	0.5393	0.7822	0.1985	0.1453	0.5648	0.6765
SNRTOI *	0.1321	0.0728	0.4094	0.6784	0.1016	0.0720	0.3169	0.8930	0.1169	0.0724	0.3632	0.7857
ENMIQA *	0.3630	0.2479	0.5093	0.5873	0.7941	0.6119	0.8313	0.5130	0.5786	0.4299	0.6703	0.5502
*R18GR50M* *	0.6299	0.5012	0.7999	0.4381	**0.8998**	**0.7398**	**0.9270**	**0.3465**	**0.7649**	**0.6205**	**0.8635**	**0.3923**
*R50GR18* *	**0.7423**	**0.6039**	**0.8206**	**0.4238**	**0.9083**	**0.7490**	**0.9294**	**0.3479**	**0.8253**	**0.6765**	**0.8750**	**0.3859**
*MR50* *	**0.7036**	**0.5740**	**0.8576**	**0.3865**	0.8919	0.7176	0.9241	0.3560	**0.7978**	**0.6458**	**0.8909**	**0.3712**

Note: * denotes the approach designed for the evaluation of MR images. The best three results for each criterion are written in bold, the names of fusion architectures introduced in this paper are written in italics.

**Table 2:** Run-time comparison. The approach designed for the evaluation of MR images is denoted by *. The names of fusion architectures introduced in this paper are written in italics.

Method	Time (s)
NOMRIQA *	0.1564
HOSA	0.2992
NOREQI	0.1315
IL-NIQE	4.4956
GM-LOG	0.0138
GWHGLBP	0.0336
BRISQUE	0.0232
SISBLIM	0.7821
metricQ	0.1994
BPRI	0.1473
SINDEX	0.0141
NFERM	9.5449
SEER	0.3473
MEON	0.0775
DEEPIQ	1.2746
SNRTOI *	0.0018
ENMIQA *	0.0737
*R18GR50M* *	0.0293
*R50GR18* *	0.0237
*MR50* *	0.0226

**Table 3:** Cross-database performance of twenty evaluated NR approaches.

Method	Training on Database 1				Training on Database 2				Overall			
	Testing on Database 2				Testing on Database 1							
	SRCC	KRCC	PLCC	RMSE	SRCC	KRCC	PLCC	RMSE	SRCC	KRCC	PLCC	RMSE
NOMRIQA *	0.7348	0.5280	0.7861	0.5979	**0.6116**	**0.4436**	0.7113	0.5116	0.6732	0.4858	0.7487	0.5548
HOSA	0.7625	0.5612	0.7968	0.5845	0.4550	0.3311	0.6428	0.5574	0.6088	0.4462	0.7198	0.5710
NOREQI	0.7259	0.5312	0.7436	0.6468	0.5082	0.3719	0.7019	0.5183	0.6171	0.4516	0.7228	0.5826
IL-NIQE	0.0050	0.0044	0.1773	0.9520	0.1796	0.1162	0.3465	0.6826	0.0923	0.0603	0.2619	0.8173
GM-LOG	0.7064	0.5134	0.7420	0.6486	0.2721	0.1774	0.1379	0.7207	0.4893	0.3454	0.4400	0.6847
GWHGLBP	0.6247	0.4315	0.6656	0.7220	0.5207	0.3694	0.6189	0.5716	0.5727	0.4005	0.6423	0.6468
BRISQUE	0.6528	0.4640	0.7294	0.6618	0.4895	0.3353	0.6172	0.5725	0.5712	0.3997	0.6733	0.6172
SISBLIM	0.6836	0.5037	0.6746	0.7140	0.2885	0.1820	0.5733	0.5962	0.4861	0.3429	0.6240	0.6551
metricQ	0.4642	0.3271	0.3931	0.8942	0.2300	0.1520	0.2243	0.7091	0.3471	0.2396	0.3087	0.8017
BPRI	0.0747	0.0558	0.4592	0.8593	0.1515	0.1120	0.3440	0.6832	0.1131	0.0839	0.4016	0.7713
SINDEX	0.2807	0.1935	0.3604	0.9024	0.2802	0.1962	0.3307	0.6869	0.2805	0.1949	0.3456	0.7947
NFERM	0.6718	0.4856	0.7240	0.6672	0.4660	0.3536	0.4637	0.6447	0.5689	0.4196	0.5939	0.6560
SEER	0.7855	0.5960	0.8356	0.5314	0.5397	0.4053	**0.7341**	**0.4941**	0.6626	0.5007	0.7849	0.5128
MEON	0.5314	0.3701	0.5148	0.8293	0.1247	0.0771	0.1401	0.7205	0.3281	0.2236	0.3275	0.7749
DEEPIQ	0.3620	0.2528	0.5778	0.7895	0.3030	0.2037	0.4041	0.6656	0.3325	0.2283	0.4910	0.7276
SNRTOI *	0.0681	0.0443	0.1033	0.9622	0.1828	0.1245	0.2262	0.7088	0.1255	0.0844	0.1648	0.8355
ENMIQA *	0.7631	0.5736	0.8040	0.5753	0.3540	0.2428	0.6741	0.5375	0.5586	0.4082	0.7391	0.5564
*R18GR50M* *	**0.8451**	**0.6574**	**0.8911**	**0.4390**	0.6098	0.4402	0.7231	0.5026	**0.7275**	**0.5488**	**0.8071**	**0.4708**
*R50GR18* *	**0.8638**	**0.6684**	**0.8930**	**0.4354**	**0.6163**	**0.4502**	**0.7306**	**0.4968**	**0.7401**	**0.5593**	**0.8118**	**0.4661**
*MR50* *	**0.8568**	**0.6709**	**0.8941**	**0.4332**	**0.6299**	**0.4686**	**0.7345**	**0.4938**	**0.7434**	**0.5697**	**0.8143**	**0.4635**

Note: * denotes the approach designed for the evaluation of MR images. The best three results for each criterion are written in bold, the names of fusion architectures introduced in this paper are written in italics.

## Data Availability

Publicly available datasets were analyzed in this study. This data can be found here: http://marosz.kia.prz.edu.pl/fusionMRIQA.html.
